# Supplementary material for: Bioprospecting the Solar Panel Microbiome: High-Throughput Screening for Antioxidant Bacteria in a Caenorhabditis elegans Model
Source: Front Microbiol. 2019 May 7;10:986. doi: 10.3389/fmicb.2019.00986 (PMC6514134; doi:10.3389/fmicb.2019.00986)
Supplement: Supplementary file 1 [file Data_Sheet_1.PDF]

Supplementary Table 1. Collection of identified bacterial isolates from solar panel surfaces. The GenBank/EMBL/DDBJ accession numbers for the partial 16S rDNA sequence of each strain is indicated, as well as the taxonomic affiliation, the closest neighbor with valid name (species, strain and accession number) and the % of similarity.

Strain	Genbank accession number	Taxonomic affiliation	Closest neighbour (accession number)	% similarity
**PS1**	MK621939	*Firmicutes;Bacilli;Bacillales;Planococcaceae;Planococcus*	*Planomicrobium glaciei* 423 (EU036220)	97.38
**PS2**	MK621940	*Actinobacteria;Actinobacteria_c;Micrococcales;Micrococcaceae;Arthrobacter*	*Arthrobacter gandavensis* R 5812 (AJ316140)	100
**PS4**	MK621941	*Proteobacteria;Gammaproteobacteria;Enterobacterales;Erwiniaceae;Pantoea*	*Pantoea septica* LMG 5345 (MLJJ01000077)	99.69
**PS5**	MK621942	*Actinobacteria;Actinobacteria_c;Micrococcales;Microbacteriaceae;Curtobacterium*	*Curtobacterium herbarum* P 420/07 (AJ310413)	98.61
**PS6**	MK621943	*Actinobacteria;Actinobacteria_c;Micrococcales;Microbacteriaceae;Curtobacterium*	*Curtobacterium citreum* DSM 20528 (X77436)	99.06
**PS7**	MK621944	*Actinobacteria;Actinobacteria_c;Micrococcales;Microbacteriaceae;Curtobacterium*	*Curtobacterium herbarum* P 420/07 (AJ310413)	98.59
**PS8**	MK621945	*Actinobacteria;Actinobacteria_c;Micrococcales;Microbacteriaceae;Frigoribacterium*	*Frigoribacterium endophyticum* EGI 6500707 (KM114212)	99.73
**PS9**	MK621946	*Actinobacteria;Actinobacteria_c;Micrococcales;Micrococcaceae;Kocuria*	*Kocuria rosea* DSM 20447 (X87756)	99.75
**PS10**	MK621947	*Actinobacteria;Actinobacteria_c;Micrococcales;Micrococcaceae;Glutamicibacter*	*Glutamicibacter arilaitensis* Re117 (FQ311875)	100
**PS13**	MK621948	*Actinobacteria;Actinobacteria_c;Micrococcales;Jonesiaceae;Sanguibacter*	*Sanguibacter inulinus* ST50 (X79451)	100
**PS14**	MK621949	*Actinobacteria;Actinobacteria_c;Micrococcales;Micrococcaceae;Kocuria*	*Kocuria rosea* DSM 20447 (X87756)	99.53
**PS16**	MK621950	*Actinobacteria;Actinobacteria_c;Micrococcales;Micrococcaceae;Kocuria*	*Kocuria rosea* DSM 20447 (X87756)	99.77
**PS17**	MK621951	*Actinobacteria;Actinobacteria_c;Micrococcales;Micrococcaceae;Arthrobacter*	*Arthrobacter pityocampae* Tp2 (EU855749)	97.77

**PS19**	MK621952	*Actinobacteria;Actinobacteria_c;Micrococcales;Jonesiaceae;Sanguibacter*	*Sanguibacter inulinus* ST50 (X79451)	100
**PS20**	MK621953	*Actinobacteria;Actinobacteria_c;Micrococcales;Microbacteriaceae;Curtobacterium*	*Curtobacterium herbarum P 420/07 (AJ310413)*	98.37
**PS21**	MK621954	*Proteobacteria;Alphaproteobacteria;Rhodobacterales;Rhodobacteraceae;Rhodobacter*	*Rhodobacter maris* JA276 (AM745438)	98.89
**PS22**	MK621955	*Actinobacteria;Actinobacteria_c;Micrococcales;Microbacteriaceae;Curtobacterium*	*Curtobacterium flaccumfaciens*LMG 3645 (AJ312209)	99.08
**PS27**	MK621956	*Actinobacteria;Actinobacteria_c;Micrococcales;Microbacteriaceae;Microbacterium*	Microbacterium imperiale DSM 20530 (X77442)	100
**PS28**	MK621957	*Actinobacteria;Actinobacteria_c;Micrococcales;Micrococcaceae;Kocuria*	*Kocuria rosea* DSM 20447 (X87756)	99.77
**PS29**	MK621958	*Actinobacteria;Actinobacteria_c;Micrococcales;Micrococcaceae;Kocuria*	*Kocuria rosea* DSM 20447 (X87756)	99.77
**PS30**	MK621959	*Actinobacteria;Actinobacteria_c;Micrococcales;Micrococcaceae;Arthrobacter*	*Arthrobacter agilis* DSM 20550 (X80748)	99.26
**PS31**	MK621960	*Actinobacteria;Actinobacteria_c;Micrococcales;Micrococcaceae;Kocuria*	*Kocuria rosea* DSM 20447 (X87756)	99.77
**PS33**	MK621961	*Proteobacteria;Alphaproteobacteria;Rhodobacterales;Rhodobacteraceae;Paracoccus*	*Paracoccus marcusii* DSM 11574 (Y12703)	99.73
**PS35**	MK621962	*Actinobacteria;Actinobacteria_c;Micrococcales;Microbacteriaceae;Curtobacterium*	*Curtobacterium flaccumfaciens* LMG 3645 (AJ312209)	99.77
**PS36**	MK621963	*Actinobacteria;Actinobacteria_c;Micrococcales;Microbacteriaceae;Curtobacterium*	*Curtobacterium herbarum* P 420/07 (AJ310413)	97.9
**PS37**	MK621964	*Actinobacteria;Actinobacteria_c;Micrococcales;Microbacteriaceae;Pseudoclavibacter*	*Pseudoclavibacter terrae* THG-MD12 (KJ769174)	99.74
**PS38**	MK621965	*Actinobacteria;Actinobacteria_c;Micrococcales;Microbacteriaceae;Microbacterium*	*Microbacterium yannicii* G72 (FN547412)	99.33
**PS39**	MK621966	*Actinobacteria;Actinobacteria_c;Micrococcales;Micrococcaceae;Arthrobacter*	*Arthrobacter agilis* DSM 20550 (X80748)	99.22

**PS40**	MK621967	*Actinobacteria;Actinobacteria_c;Micrococcales;Microbacteriaceae;Microbacterium*	*Microbacterium imperiale* DSM 20530 (X77442)	99.32
**PS41**	MK621968	*Actinobacteria;Actinobacteria_c;Micrococcales;Microbacteriaceae;Microbacterium*	*Microbacterium arborescens* DSM 20754 (X77443)	99.5
**PS42**	MK621969	*Actinobacteria;Actinobacteria_c;Micrococcales;Microbacteriaceae;Microbacterium*	*Microbacterium oleivorans* NBRC 103075 (BCRG01000019)	100
**PS43**	MK621970	*Actinobacteria;Actinobacteria_c;Micrococcales;Microbacteriaceae;Plantibacter*	*Plantibacter flavus* VKM Ac-2504 (jgi.1118344)	98.45
**PS44**	MK621971	*Actinobacteria;Actinobacteria_c;Micrococcales;Microbacteriaceae;Microbacterium*	*Microbacterium foliorum* DSM12966 (JYIU01000006)	99.44
**PS46**	MK621972	*Actinobacteria;Actinobacteria_c;Micrococcales;Micrococcaceae;Kocuria*	*Kocuria rosea* DSM 20447 (X87756)	99.75
**PS47**	MK621973	*Actinobacteria;Actinobacteria_c;Micrococcales;Micrococcaceae;Arthrobacter*	*Arthrobacter subterraneus* CH7 (DQ097525)	97.95
**PS48**	MK621974	*Proteobacteria;Gammaproteobacteria;Pseudomonadales;Pseudomonadaceae;Pseudomonas*	*Pseudomonas flavescens* LMG 18387 (FNDG01000047)	99.35
**PS52**	MK621975	*Actinobacteria;Actinobacteria_c;Micrococcales;Micrococcaceae;Arthrobacter*	*Arthrobacter ruber* MDB1-42 (JX949648)	98.71
**PS53**	MK621976	*Bacteroidetes;Cytophagia;Cytophagales;Hymenobacteraceae;Pontibacter*	*Pontibacter saemangeumensis* GCM0142 (JN607163)	98.2
**PS54**	MK621977	*Proteobacteria;Alphaproteobacteria;Sphingomonadales;Sphingomonadaceae;Sphingomonas*	*Sphingomonas phyllosphaerae* FA2 (KE386571)	100
**PS55**	MK621978	*Actinobacteria;Actinobacteria_c;Micrococcales;Micrococcaceae;Kocuria*	*Kocuria rosea* DSM 20447 (X87756)	99.56
**PS56**	MK621979	*Actinobacteria;Actinobacteria_c;Micrococcales;Micrococcaceae;Kocuria*	*Kocuria rosea* DSM 20447 (X87756)	99.77
**PS57**	MK621980	*Proteobacteria;Alphaproteobacteria;Sphingomonadales;Sphingomonadaceae;Sphingomonas*	*Sphingomonas aerolata* NW12 (AJ429240)	99.76
**PS58**	MK621981	*Actinobacteria;Actinobacteria_c;Micrococcales;Microbacteriaceae;Microbacterium*	*Microbacterium terregens* IFO 12961 (AB004721)	97.47

**PS59**	MK621982	*Actinobacteria;Actinobacteria_c;Micrococcales;Microbacteriaceae;Microbacterium*	*Microbacterium radiodurans* GIMN1.002 (GQ329713)	100
**PS60**	MK621983	*Proteobacteria;Alphaproteobacteria;Sphingomonadales;Sphingomonadaceae;Sphingomonas*	*Sphingomonas aerolata* NW12 (AJ429240)	100
**PS62**	MK621984	*Actinobacteria;Actinobacteria_c;Micrococcales;Promicromonosporaceae;Cellulosimicrobium*	*Cellulosimicrobium cellulans* LMG 16121 (CAOI01000359)	100
**PS63**	MK621985	*Actinobacteria;Actinobacteria_c;Micrococcales;Micrococcaceae;Arthrobacter*	*Arthrobacter agilis* DSM 20550 (X80748)	98.87
**PS64**	MK621986	*Proteobacteria;Alphaproteobacteria;Sphingomonadales;Sphingomonadaceae;Sphingomonas*	*Sphingomonas endophytica* YIM 65583 (HM629444)	99.75
**PS65**	MK621987	*Bacteroidetes;Sphingobacteriia;Sphingobacteriales;Sphingobacteriaceae;Pedobacter*	*Pedobacter agri* PB92 (AJLG01000244)	98.23
**PS66**	MK621988	*Actinobacteria;Actinobacteria_c;Micrococcales;Microbacteriaceae;Agrococcus*	*Agrococcus citreus* IAM 15145 (AB279547)	99.7
**PS67**	MK621989	*Proteobacteria;Alphaproteobacteria;Rhodobacterales;Rhodobacteraceae;Paracoccus*	*Paracoccus aestuarii* B7 (EF660757)	97.6
**PS68**	MK621990	*Actinobacteria;Actinobacteria_c;Micrococcales;Promicromonosporaceae;Cellulosimicrobium*	*Cellulosimicrobium funkei* LMG 16121 (CAOI01000359)	100
**PS69**	MK621991	*Actinobacteria;Actinobacteria_c;Micrococcales;Microbacteriaceae;Microbacterium*	*Microbacterium oleivorans* NBRC 103075 (BCRG01000019)	100
**PS70**	MK621992	*Actinobacteria;Actinobacteria_c;Micrococcales;Micrococcaceae;Kocuria*	*Kocuria rosea* DSM 20447 (X87756)	99.73
**PS71**	MK621993	*Actinobacteria;Actinobacteria_c;Micrococcales;Microbacteriaceae;Leucobacter*	*Leucobacter chromiiresistens* JG31 (AGCW01000231)	99.04
**PS72**	MK621994	*Actinobacteria;Actinobacteria_c;Micrococcales;Microbacteriaceae;Curtobacterium*	*Curtobacterium citreum* DSM 20528 (X77436)	99.25
**PS74**	MK621995	*Actinobacteria;Actinobacteria_c;Micrococcales;Micrococcaceae;Kocuria*	*Kocuria polaris* CMS 76or (JSUH01000031)	99.47
**PS75**	MK621996	*Firmicutes;Bacilli;Bacillales;Bacillaceae;Bacillus*	*Bacillus megaterium* NBRC 15308 (JJMH01000057)	100

**PS76**	MK621997	*Firmicutes;Bacilli;Bacillales;Bacillaceae;Bacillus*	*Bacillus endophyticus* 2DT (AF295302)	99.34
**PS78**	MK621998	*Firmicutes;Bacilli;Bacillales;Bacillaceae;Bacillus*	*Bacillus endophyticus* 2DT (AF295302)	99.13
**PS79**	MK621999	*Firmicutes;Bacilli;Bacillales;Bacillaceae;Bacillus*	*Bacillus atrophaeus* JCM 9070 (AB021181)	99.58
**PS80**	MK622000	*Firmicutes;Bacilli;Bacillales;Bacillaceae;Bacillus*	*Bacillus endophyticus* 2DT (AF295302)	99.55
**PS81**	MK622001	*Firmicutes;Bacilli;Bacillales;Bacillaceae;Bacillus*	*Bacillus megaterium* NBRC 15308 (JJMH01000057)	99.38
**PS82**	MK622002	*Firmicutes;Bacilli;Bacillales;Bacillaceae;Bacillus*	*Bacillus atrophaeus* JCM 9070 (AB021181)	99.56
**PS83**	MK622003	*Firmicutes;Bacilli;Bacillales;Bacillaceae;Bacillus*	*Bacillus aryabhattai* B8W22 (EF114313)	99.77
**PS84**	MK622004	*Actinobacteria;Actinobacteria_c;Micrococcales;Micrococcaceae;Arthrobacter*	*Arthrobacter ruber* MDB1-42 (JX949648)	99.29
**PS85**	MK622005	*Actinobacteria;Actinobacteria_c;Micrococcales;Micrococcaceae;Kocuria*	*Kocuria rosea* DSM 20447 (X87756)	99.54
**PS86**	MK622006	*Actinobacteria;Actinobacteria_c;Micrococcales;Micrococcaceae;Arthrobacter*	*Arthrobacter pityocampae* Tp2 (EU855749)	99.38
