# Supplementary material for: Bioprospecting the Solar Panel Microbiome: High-Throughput Screening for Antioxidant Bacteria in a Caenorhabditis elegans Model
Source: Front Microbiol. 2019 May 7;10:986. doi: 10.3389/fmicb.2019.00986 (PMC6514134; doi:10.3389/fmicb.2019.00986)
Supplement: Supplementary file 2 [file Data_Sheet_2.PDF]

Supplementary Table 2. Carotenoids (CRTs) identified in the solar panel isolates in liquid (L) and solid (S) cultures. Numbers correspond to the chromatographic peaks represented in Supplementary Figure 2. Maximum wavelengths and %III/II (numeric indication of the spectral shape, specifically the relationship between the peak heights of the longest-wavelength absorption band – III – and the middle absorption band – II -, expressed as a percentage) are represented and compared with data from the literature (Britton et al., 1998; Rodrigo et al., 2003). In the final column, quantification is displayed in micrograms of each CRT per gram of cellular pellet (dry pellet in the stationary phase samples and wet pellet in the exponential phase samples). Non-identified CRTs are indicated as “NI”; s, denotes shoulder in the spectrum; *CRTs were tentatively identified (for which a standard was not available).

		Observed			Literature		
**Isolate**		**Carotenoid**	**λ_{max} (nm)**	**% III/II**	**λ_{max} (nm)**	**% III/II**	**µg/g**
PS1 - L	1	NI-1	440,467,498	17.5	-	-	0,2
	2	Total phytoene	278,285,301	14	276, 286, 297	10	16,9
	3	Total phytofluene	322,346,362	69	331, 348, 367	90	0,3
	4	β-cryptoxanthin	426,453,482	27	428, 450, 478	25	0,02
PS1 - S	5	Total phytoene	278,285,301	15	276, 286, 297	10	197,5
	6	Phytofluene	322,347,364	78	331, 348, 367	90	2,8
	7	NI-2	cis355,s,458,s	-	-	-	9,1
	8	NI-3	cis355,s,458,s	-	-	-	10,6
	9	β-cryptoxanthin	422,454,480	31	428, 450, 478	25	54,8
PS21 - L	10	Adonirubin*	473	-	474	-	6,4
	11	Canthaxanthin	464	-	466	-	12,0
	12	Phytoene	278,285,301	10	276, 286, 297	10	9,4
PS21 - S	13	Astaxanthin*	474	-	474	-	13,4
	14	Adonirubin*	472	-	474	-	11,9
	15	Canthaxanthin	463	-	466	-	63,9
	16	Phytoene	278,285,301	11	276, 286, 297	10	7,8
	17	Echinenone*	466	-	461	-	0,4
	18	β-carotene*	425,452,581	33	425, 450, 477	25	8,9
PS75 - L	19	NI-4	429,454, 475	75			0,03
	20	NI-5	,429,454,475	71			0,04
PS75 - S	21	NI-6	378,402,427	134	-	-	0,4
	22	Mix Phytoene-like *;and NI-7	278,285,301; s,363,390	<1	274, 286, 298	<1	mix
	23	NI-8	376,389,422	73		90	0,2
	24	NI-9	373,402,427	86	-	-	2,2
	25	NI-10	378,402,425	100	-	-	1,9
	26	NI-11	380,417,445	87	-	-	0,9
	27	NI-12	391,416,445	126	-	-	0,5
	28	NI-13	394,419,445	67	-	-	2,1
	29	NI-14	cis341,422,446,475	52			1,3
	30	NI-15	cis343,422,447,476	69			0,6
	31	NI-16	423,447,477	76			0,7
	32	NI-17	443,453,484	81			12,6
