# Supplementary material for: Bioprospecting the Solar Panel Microbiome: High-Throughput Screening for Antioxidant Bacteria in a Caenorhabditis elegans Model
Source: Front Microbiol. 2019 May 7;10:986. doi: 10.3389/fmicb.2019.00986 (PMC6514134; doi:10.3389/fmicb.2019.00986)
Supplement: Supplementary file 3 [file Data_Sheet_3.PDF]

Supplementary Figure 1. HPLC chromatograms of the pigments extracted from the solar panel isolates grown in liquid and solid medium. Above, the MaxPlot chromatogram is represented, and the peaks of interest are numbered in accordance with the carotenoids described in Table 1. Spectra of peak of interests are represented below the MaxPlot chromatogram. **(A)** PS1 in liquid medium. **(B)** PS1 in solid medium. **(C)** PS21 in liquid medium. **(D)** PS21 in solid medium. **(E)** PS75 in liquid medium. **(F)** PS75 in solid medium.

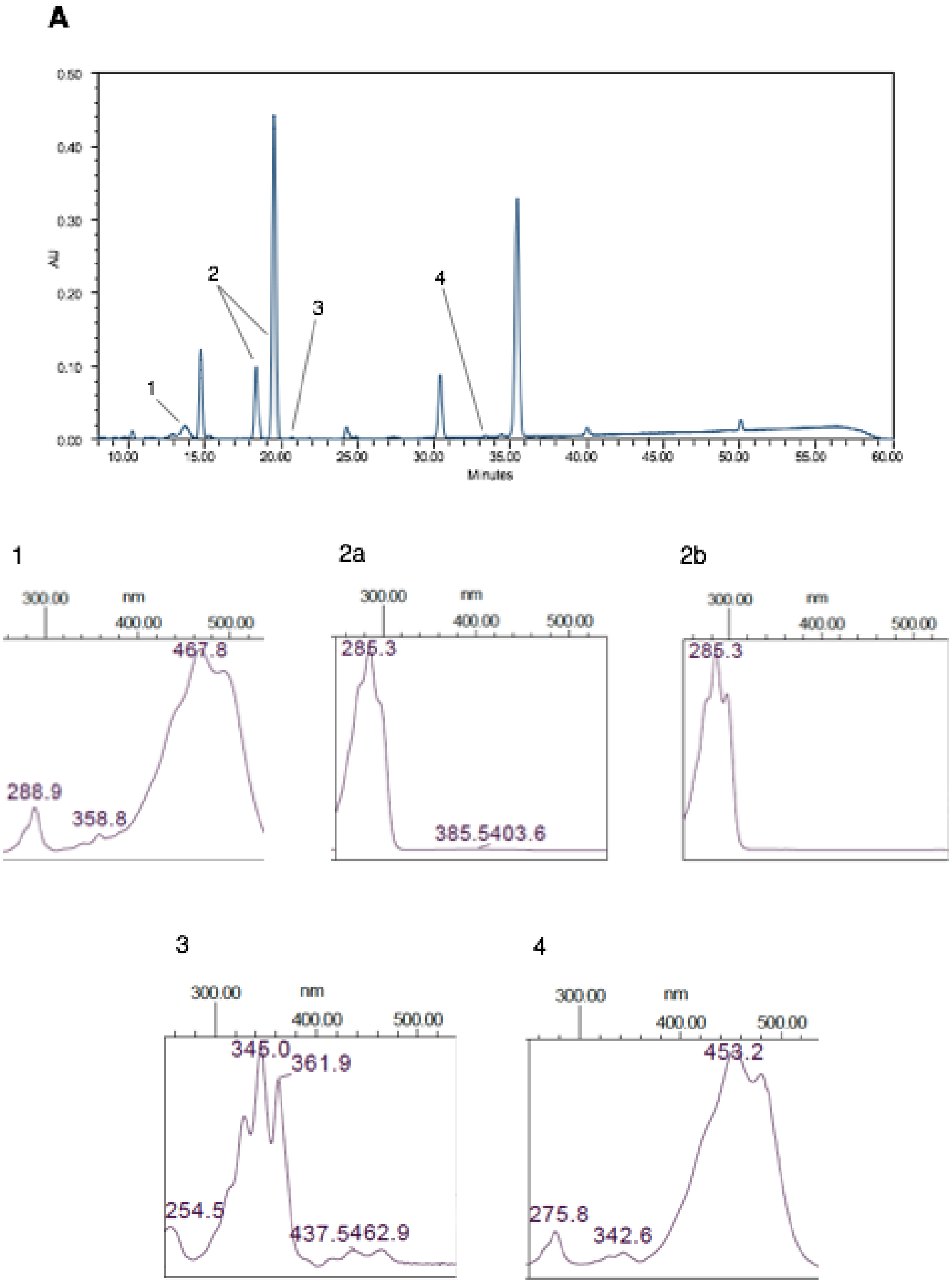

B

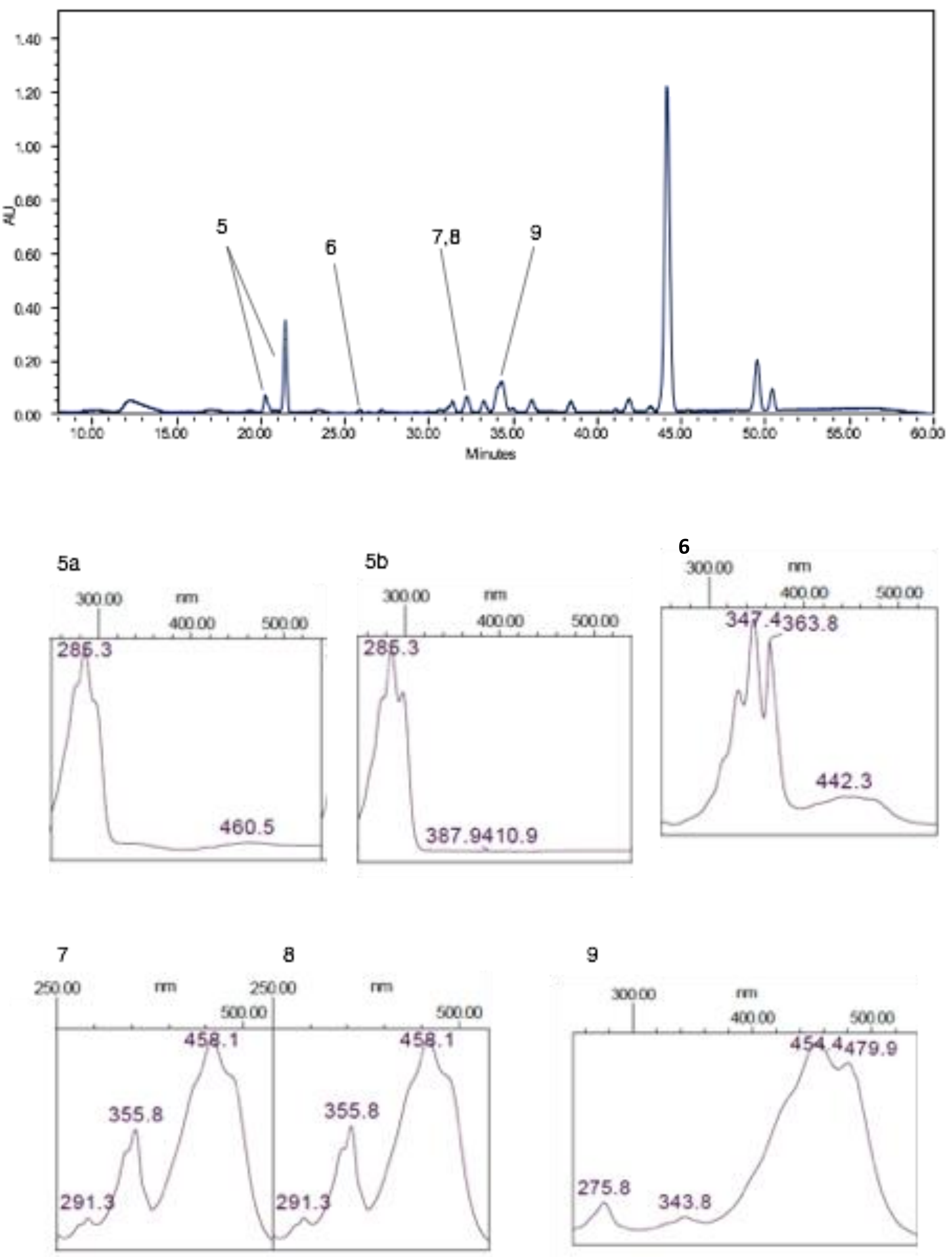

AU
1.40
1.20
1.00
0.80
0.60
0.40
0.20
0.00
5
6
7,8
9
10.00
15.00
20.00
25.00
30.00
35.00
40.00
45.00
50.00
55.00
60.00
Minutes
5a
300.00
nm
400.00
500.00
285.3
460.5
5b
300.00
nm
400.00
500.00
285.3
387.9410.9
6
300.00
nm
400.00
500.00
347.4363.8
442.3
7
250.00
nm
500.00
458.1
355.8
291.3
8
250.00
nm
500.00
458.1
355.8
291.3
9
300.00
nm
400.00
500.00
454.4479.9
275.8
343.8

C

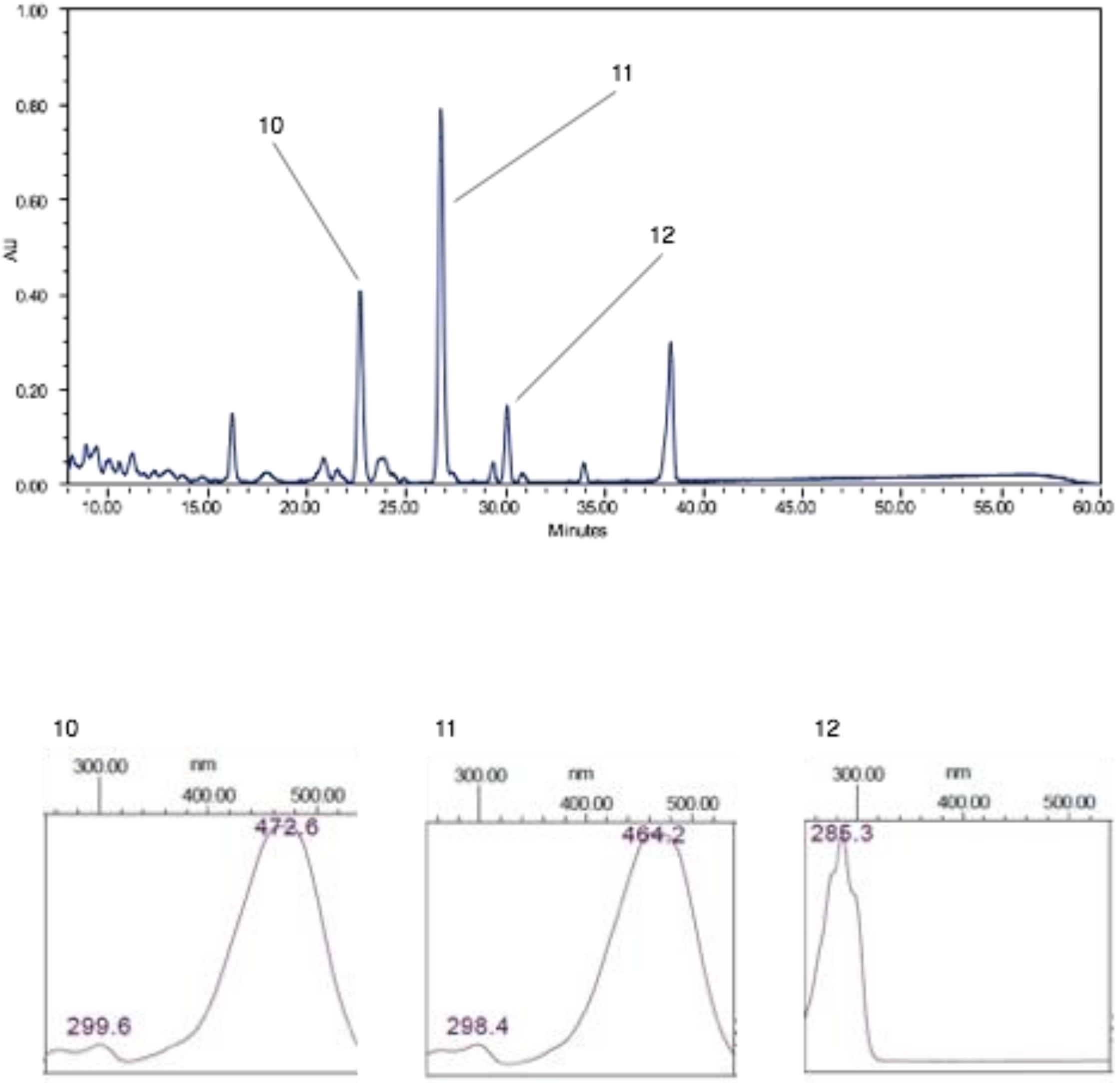
1.00
0.80
0.60
0.40
0.20
0.00
AU
10.00
15.00
20.00
25.00
30.00
35.00
40.00
45.00
50.00
55.00
60.00
Minutes
10
11
12
10
300.00
nm
400.00
500.00
472.6
299.6
11
300.00
nm
400.00
500.00
464.2
298.4
12
300.00
nm
400.00
500.00
285.3

D

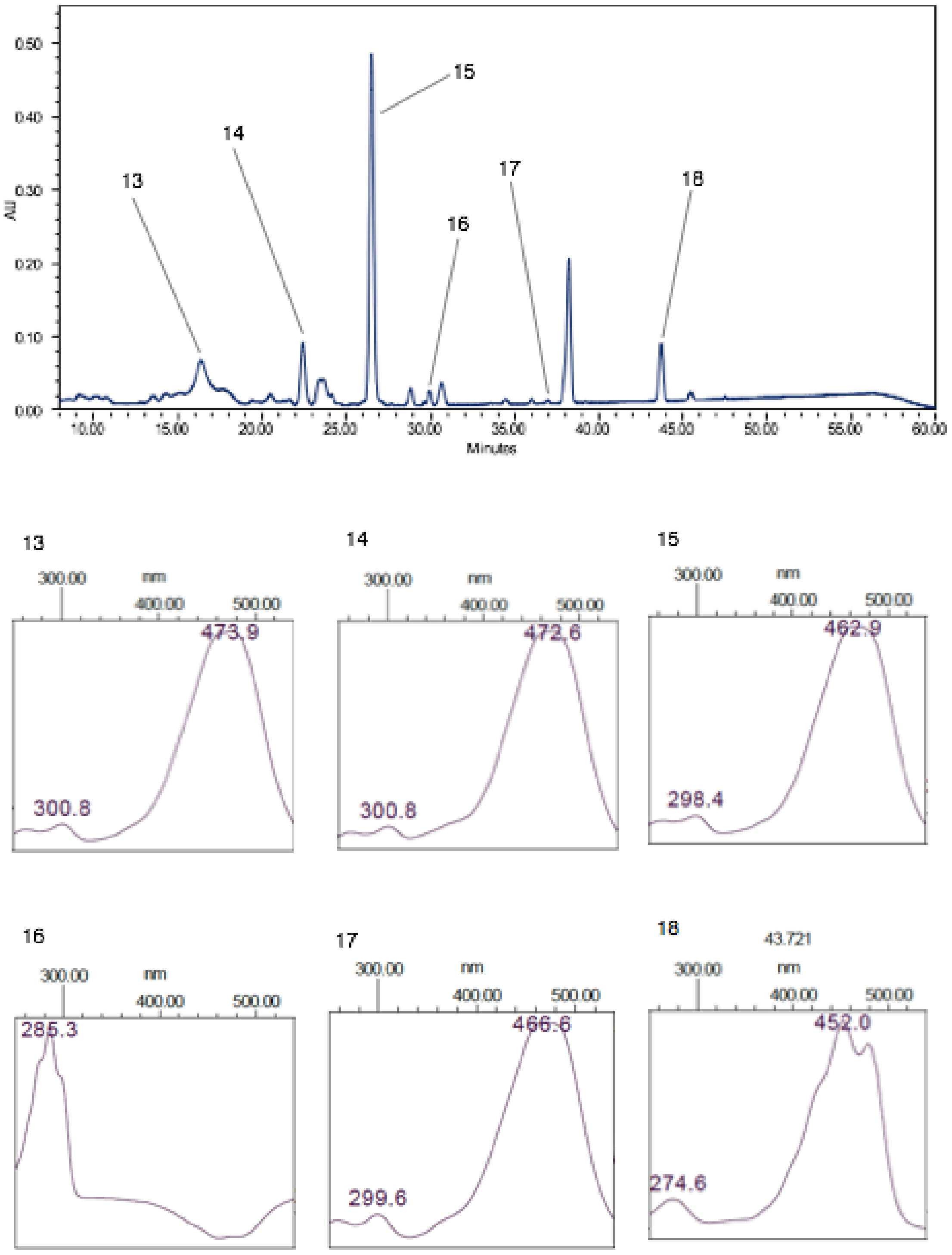
AU
0.00
0.10
0.20
0.30
0.40
0.50
10.00
15.00
20.00
25.00
30.00
35.00
40.00
45.00
50.00
55.00
60.00
Minutes
13
14
15
16
17
18
13
300.00
nm
400.00
500.00
473.9
300.8
14
300.00
nm
400.00
500.00
472.6
300.8
15
300.00
nm
400.00
500.00
462.9
298.4
16
300.00
nm
400.00
500.00
285.3
17
300.00
nm
400.00
500.00
466.6
299.6
18
43.721
300.00
nm
400.00
500.00
452.0
274.6

E

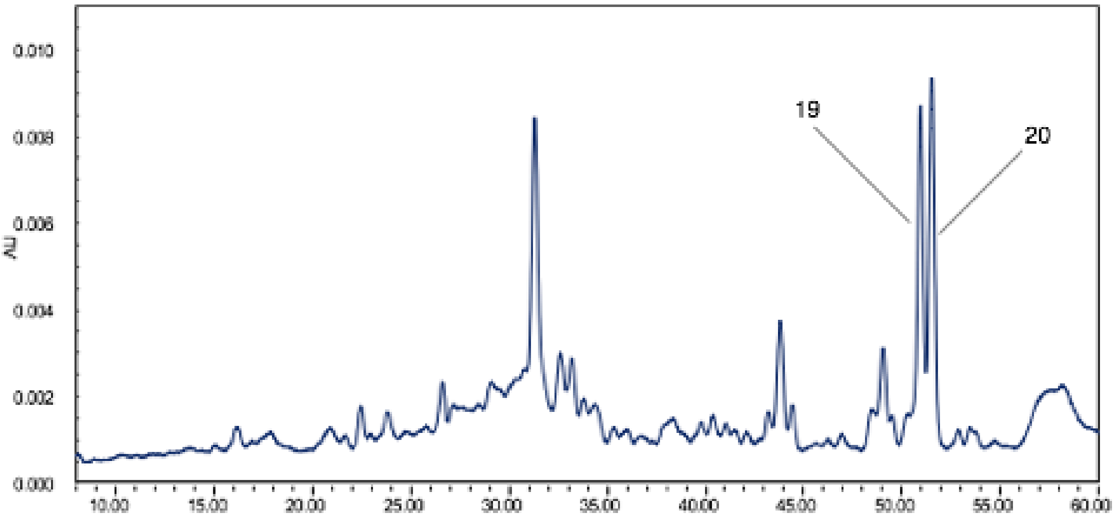
AU
0.000
0.002
0.004
0.006
0.008
0.010
10.00
15.00
20.00
25.00
30.00
35.00
40.00
45.00
50.00
55.00
60.00
19
20

19

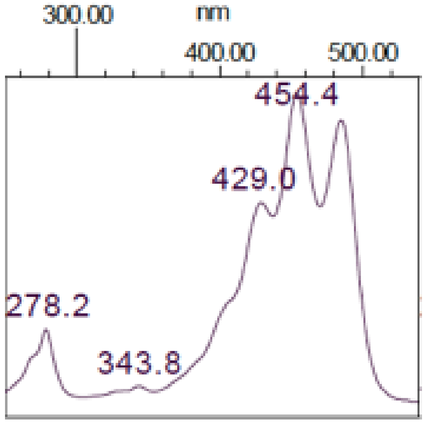
300.00
nm
400.00
500.00
454.4
429.0
278.2
343.8

20

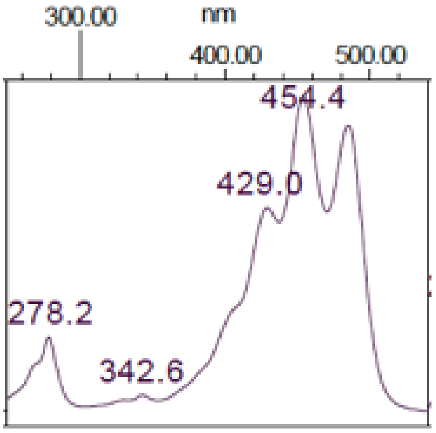
300.00
nm
400.00
500.00
454.4
429.0
278.2
342.6

F

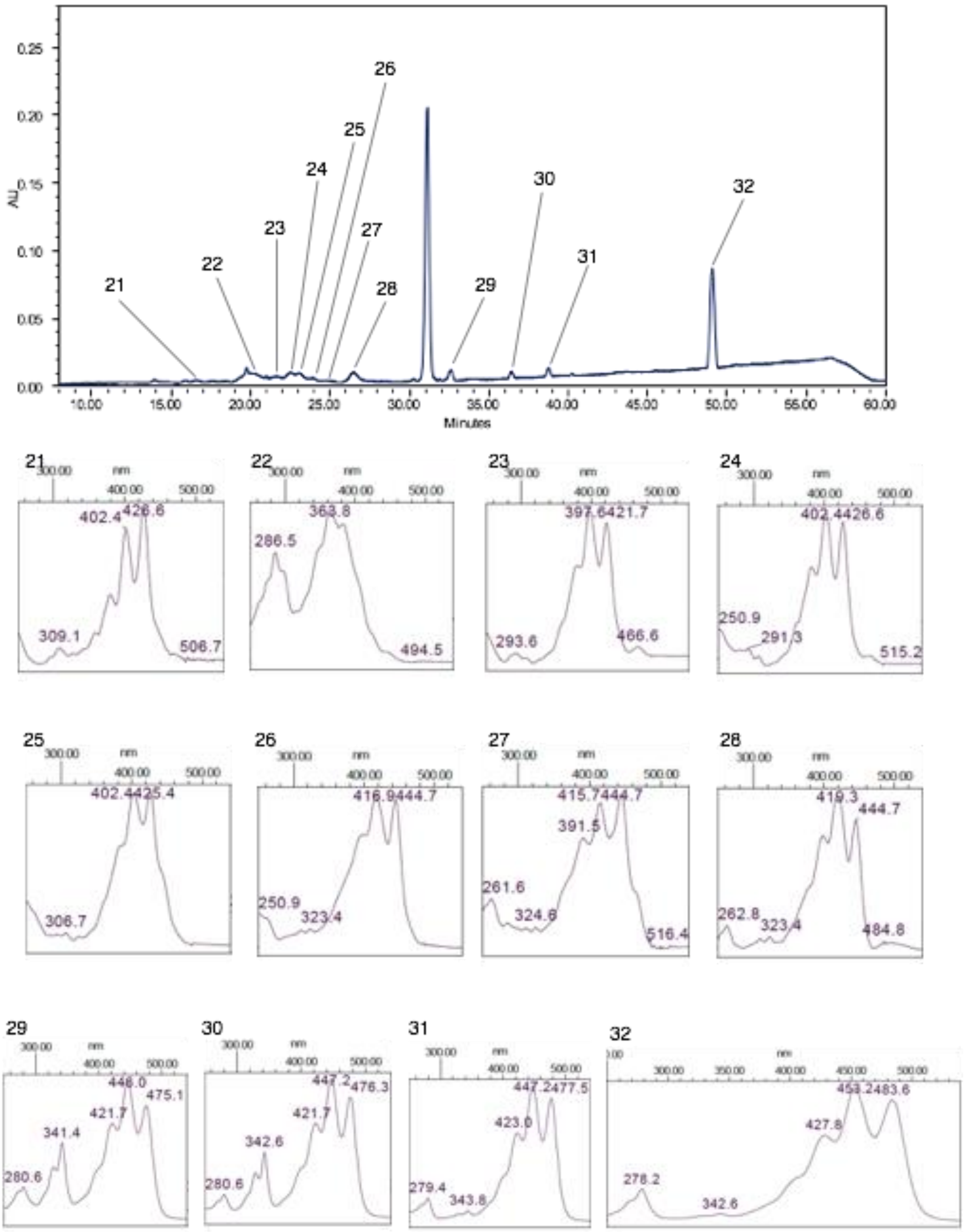

AU
0.25
0.20
0.15
0.10
0.05
0.00
10.00
15.00
20.00
25.00
30.00
35.00
40.00
45.00
50.00
55.00
60.00
Minutes
21
22
23
24
25
26
27
28
29
30
31
32
21 nm 300.00 400.00 500.00 402.4 426.6 309.1 506.7
22 nm 300.00 400.00 500.00 363.8 286.5 494.5
23 nm 300.00 400.00 500.00 397.6421.7 293.6 466.6
24 nm 300.00 400.00 500.00 402.4426.6 250.9 291.3 515.2
25 nm 300.00 400.00 500.00 402.4425.4 306.7
26 nm 300.00 400.00 500.00 416.9444.7 250.9 323.4
27 nm 300.00 400.00 500.00 415.7444.7 391.5 261.6 324.6 516.4
28 nm 300.00 400.00 500.00 419.3 444.7 262.8 323.4 484.8
29 nm 300.00 400.00 500.00 446.0 475.1 421.7 341.4 280.6
30 nm 300.00 400.00 500.00 447.2 476.3 421.7 342.6 280.6
31 nm 300.00 400.00 500.00 447.2477.5 423.0 279.4 343.8
32 nm 300.00 350.00 400.00 450.00 500.00 453.2483.6 427.8 278.2 342.6
