# Supplementary material for: Bioprospecting the Solar Panel Microbiome: High-Throughput Screening for Antioxidant Bacteria in a Caenorhabditis elegans Model
Source: Front Microbiol. 2019 May 7;10:986. doi: 10.3389/fmicb.2019.00986 (PMC6514134; doi:10.3389/fmicb.2019.00986)
Supplement: Supplementary file 4 [file Data_Sheet_4.PDF]

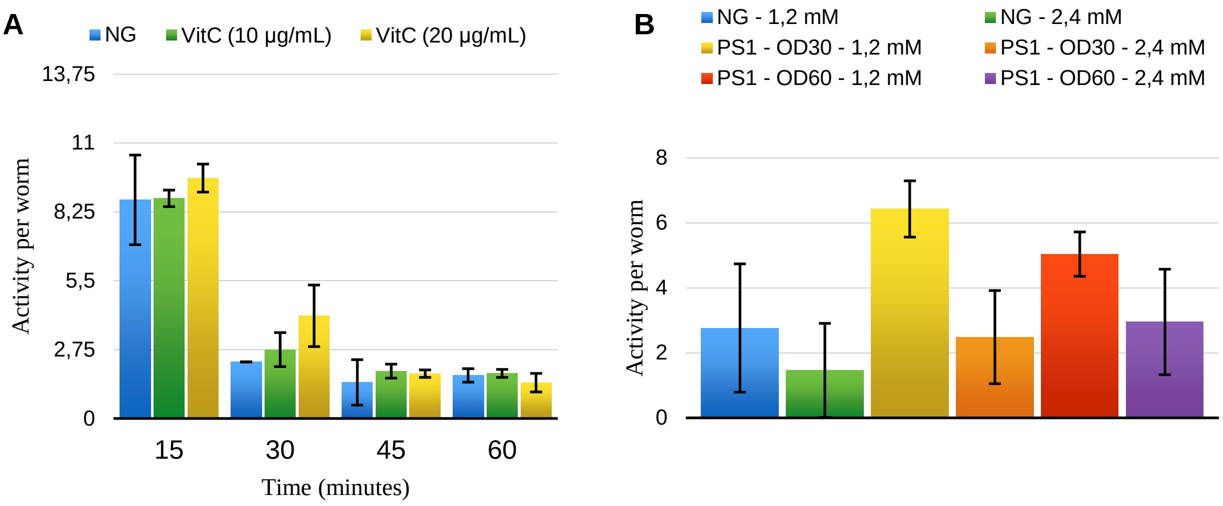

Supplementary Figure 2. Optimization of vitamin C and hydrogen peroxide concentrations for WormTracker assays. **(A)** Optimization of vitamin C concentration as a statistically significant positive control for WormTracker assays. Vitamin C was tested at 10 and at 20 µg/mL, resulting in significant differences in comparison to the negative control (NG) at 20 µg/mL. **(B)** Optimization of hydrogen peroxide concentrations for WormTracker assays. Hydrogen peroxide was tested at 1.2 and 2.4 mM, and activity per worm after 30 minutes of the negative control (NG) and of a positive control (PS1) can be seen in the histogram. As a result, 1.2 mM of hydrogen peroxide was selected as the optimum concentration for these assays.
